# Short Immobilization in a Sling Does Not Lead to Increased Salivary Cortisol Levels in Pigs

**DOI:** 10.3390/ani14192760

**Published:** 2024-09-24

**Authors:** Sara Puy, Marta Giral, Dolores C. García-Olmo

**Affiliations:** 1 Centre de Recerca Experimental Biomèdica Aplicada (CREBA), IRBLleida, 25138 Lleida, Spain; spuy@creballeida.org; 2 Animal Research Facilities, Almirall SA, 08980 Barcelona, Spain; marta.giral@almirall.com

**Keywords:** cortisol, saliva, pig, restraint sling

## Abstract

**Simple Summary:**

One of the main concerns of veterinarians and researchers involved in animal research work is the improvement of experimental procedures to minimize discomfort to the animal. In the handling of experimental pigs, one of the challenges is immobilization, since due to their size, it is difficult and can cause stress to the animals. Immobilization using a restraint sling is a safe method for the animal and relatively simple for the operators. For this reason, the objective of this study was to evaluate the stress of pigs immobilized with a sling by quantifying salivary cortisol levels. We found that salivary cortisol levels did not increase when pigs were lifted and briefly restrained in the sling, even though some pigs (especially hybrids) showed apparent signs of stress. The lack of correlation between such apparent stress and salivary cortisol levels could be because the vocalizations and movements were not really signs of stress, but simply a way of releasing discomfort, learned in the process of socialization and habituation. In light of this unexpected conclusion, further studies collecting other physiological and behavioral data are needed to clarify what actually happens when pigs are restrained in a sling.

**Abstract:**

The goal of the present study was to evaluate the potential stress developed in farm hybrid pigs and miniature laboratory pigs briefly restrained in a sling, by measuring salivary cortisol levels. The study was performed in 20 healthy pigs grouped into three groups: group HYB-F: hybrid female pigs (n = 12), housed at the CREBA facility (Lleida, Spain); group MIN-F: Specipig^®^ miniature female pigs (n = 4), housed at the CREBA facility; group MIN-M: Specipig^®^ miniature male pigs (n = 4), housed at the Almirall facility (Barcelona, Spain). Upon arrival, the animals were enrolled in a social habituation and training program, which included habituation to a restraint sling. The sling was a stainless steel structure with a canvas hammock which had four openings for placing the animal’s feet. The assessment of stress levels in the sling was carried out by measuring cortisol levels in saliva samples. Five saliva samples were collected from each animal over 4 days: Sample 1 (basal sample): taken after animals perceived the presence of the technicians in the pen; Sample 2: taken after animals saw the sling in the pen; Sample 3: taken when animals were in the sling; Sample 4: taken 1 min after the previous one; Sample 5: taken after animals were released back on the floor. In group HYB-F, five animals (5/12) showed strong resistance and could not be restrained in the sling on at least one day. All animals in the groups of miniature pigs could be restrained on all the days. Within each group, the manipulation phase did not affect salivary cortisol levels. Likewise, salivary cortisol levels did not change significantly across days in either group. In conclusion, salivary cortisol levels did not increase when pigs were lifted and briefly restrained in the sling, even though some of them (in particular, the hybrid pigs) showed apparent signs of stress. The lack of correlation between such apparent stress and salivary cortisol levels might be because the vocalizations and movements were not really signs of stress, but simply a way of releasing discomfort, learned in the process of socialization and habituation. In light of this unexpected conclusion, further studies are needed to collect other physiological and behavioral data to clarify what actually happens when pigs are restrained in a sling.

## 1. Introduction

When animals are confronted with an emotional or physical stressor, several biological mechanisms are activated to elicit responses capable of coping with the homeostatic changes that occur. The two main mechanisms are the sympathetic–adrenal and hypothalamic–pituitary–adrenocortical (HPA) axes, and the main types of hormones released by these stress axes are catecholamines (CAs) and glucocorticoids (GCs), respectively. These hormones are usually determined in plasma samples as parameters of adrenal activity and, thus, of disturbance or impairment. GCs (and CAs) are extensively metabolized and subsequently excreted. Therefore, the concentration of GCs (or their metabolites) can be measured in various body fluids or excreta, such as saliva (reviewed in [1,2]).

The main GC released by the adrenal cortex after the activation of the HPA axis in pigs is cortisol [3,4]. Plasma cortisol passes into saliva in a few minutes (reviewed in [1,5]), so the use of this fluid represents a great advantage over plasma measurement, since obtaining samples from saliva is simpler, less invasive, and less stressful for the animal. Moreover, unlike plasma cortisol, which reflects both protein-bound and free fractions, salivary cortisol reflects only the free cortisol [6].

On the other hand, in both farm and laboratory environments, restraint methods are important to properly immobilize pigs for various procedures (administration of substances, blood sampling, veterinary cures, etc.). Various methods of physical restraint have been used classically, such as lifting the animal by the front or back limbs (in piglets or young animals) or by using a nose snare. Since the pig is a species very prone to elicit a stress response, it is very important to perform restraint procedures correctly and as quickly as possible.

Most of the methods mentioned above may produce stress in the animals, to a greater or lesser degree, and such stress has been assessed in some cases by measuring cortisol in saliva [7]. The use of restraint slings can be performed after habituation of the animals; it would seem to be a theoretically less stressful method for pigs. The maneuver of raising and keeping the animals in the sling for a short time is widely used to extract biologic samples for research purposes and/or to perform diverse routine manipulations on laboratory pigs (ear cleaning, nail clipping, etc.). The restraint slings are made of canvas or heavy cotton, with four padded openings for the legs, and are available in several sizes to accommodate a wide range of animals. Some authors have suggested that the pressure exerted on the abdomen of the pig has a relaxing effect [8]. However, to our knowledge, no studies have been conducted to test the stressful effect of the maneuver of immobilizing pigs in a sling for a short period.

The goal of the present study was to evaluate the potential stress developed in farm hybrid and miniature pigs briefly restrained in a sling, by measuring salivary cortisol levels.

## 2. Materials and Methods

The current study was performed in two Spanish Institutions: CREBA-IRBLleida (Lleida) and Almirall SA (Barcelona). It was approved by the Institutional Animal Care and Use Welfare Committee of CREBA (ref. PI19-004) and by the Catalan authority (ref. 10695) and was conducted in accordance with European and Spanish laws (Directive 2010/63/UE and Spanish Royal Decree 53/2013, respectively).

### 2.1. Animals and Housing Conditions

The study was performed in 20 healthy pigs, which were grouped into three groups:

- Group HYB-F: hybrid female pigs (n = 12), 3–4 months old at the beginning of the experimental procedure, purchased from an authorized provider (Centre d’Estudis Porcins, Torrelameu, Lleida, Spain). These pigs were housed at CREBA facility (Torrelameu, Lleida, Spain).
- Group MIN-F: Specipig^®^ miniature female pigs (n = 4), 7 months old at the beginning of the experimental procedure, provided by Specipig SL (El Prat de Llobregat, Barcelona, Spain), and housed at CREBA facility.
- Group MIN-M: Specipig^®^ miniature male pigs (n = 4), 10.5 months old at the beginning of the experimental procedure, provided by Specipig S.L., and housed at Almirall facility. The animals were castrated at the breeder facility and allowed to completely recover from surgery before being transported to Almirall.

Upon arrival at both CREBA and Almirall facilities, animals were evaluated by the respective designated veterinarians and were allowed to acclimate to the facility for at least 2 weeks. In animals in groups HYB-F and MIN-F (CREBA facility), the study started just after this acclimation period. Animals in group MIN-M were housed at Almirall facility for 6.5 months and were previously trained for a variety of innocuous procedures before they were used for the present study.

Only animals without any sign suggesting the presence of pathologies were included in the study.

In both centers, the pigs were housed in pens with automatic control system for temperature and ventilation, slated floor, and bowl-type drinkers. Environmental conditions were identical in both facilities, and both were in accordance with European and Spanish legislation (Directive 2010/63/UE and Spanish Royal Decree 53/2013, respectively).

The pigs had free access to water through an automatic dispensing system and were fed with standard diet, restricted to 3% of body weight, which was provided once a day. In CREBA, the diet was provided by Agrovilanova (Vilanova de la Barca, Lleida, Spain). In Almirall, it was provided by Pinallet S.A. (Cardona, Barcelona, Spain).

### 2.2. Training Procedure

Upon arrival, animals were enrolled in a habituation and training program, which was specific to each facility. Both programs started with a period of habituation to human presence and physical contact, as well as simple procedures, such as self-weighing.

**Figure 1:**
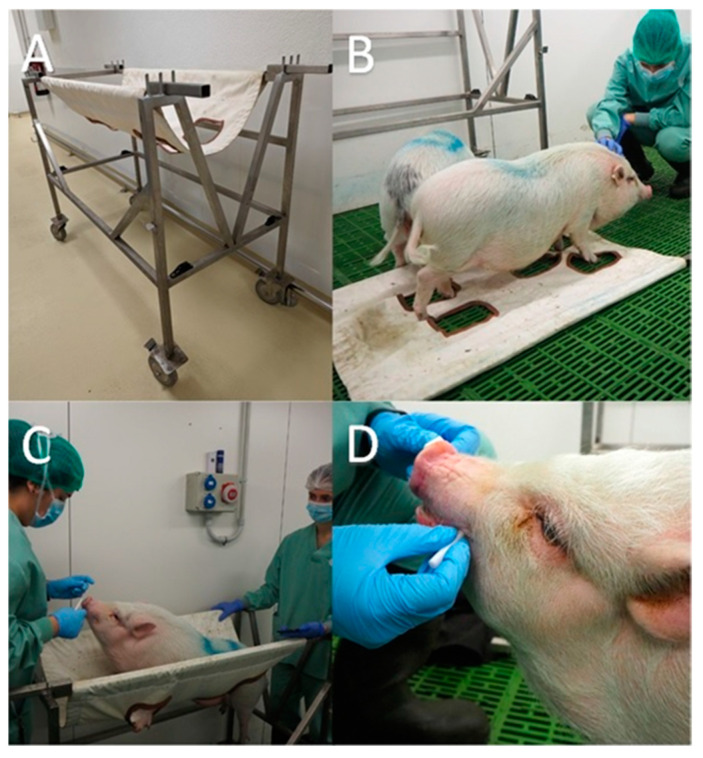
Images of some elements and moments of the experimental procedure. (**A**) Restraint sling. (**B**) The sling placed on the floor of the pen to habituate and train the pigs to be restrained. (**C**) One pig in the sling; meanwhile, the operator is taking a salivary sample. (**D**) Sampling after descending the animal from the restraining sling.

Then, the habituation to the restraining sling was started. The sling was identical in both facilities and consisted of a stainless steel structure with a canvas hammock (Harvard Apparatus Inc., Holliston, MA, USA). The canvas had four openings for placing the animal’s feet; all openings were edged in cotton jersey for comfort. The canvas was mounted on two supporting bars that made it easy to lift the animal up off the ground and lower it back down to the ground (Figure 1A).

The sling training of the animals at the CREBA facility (groups HYB-F and MIN-F) was conducted up to 13 non-consecutive days as follows:

Early in the morning, the animals were fed half ration, and after 2 h, the training was carried out. By doing this, we tried to avoid anxiety due to hunger. Once the training was over, they were given the remaining half ration.

Individual training was carried out in the housing room, without other companions or enrichment material, to avoid distractions. In the case of animals that seemed to be very stressed by being alone (shown by vocalizations and/or restless movements), exercises were performed in pairs.

A training stick and a clicker were used for this habituation, and at least two people were needed. During the process, sugar and apple pieces were used as rewards, which were gradually withdrawn, so that on the days when saliva samples were obtained for cortisol quantification, the food rewards were given at the end of the procedure, so as not to interfere with the analysis techniques.

The training procedure consisted of three progressive steps. That is, when an animal achieved the expected goal for one step, it moved on to the second step the next day. If it did not, the animal repeated the same step until it achieved it.

First step: Placement of the canvas part of the restrainer (with the supporting bars attached) on the floor to allow the animal to explore the novel object. Using a training stick, the animal was guided into the desired position on top of the canvas. When the animal showed no fear and was confident with the new object, it was rewarded with caresses, voices of encouragement, and lumps of sugar, all associated with the clicker (Figure 1B).

Second step: Placement of the limbs in the openings of the canvas. Once the animal stayed on top of the canvas, the technicians tried to have the animal place its limbs into the openings. When the animal was positioned as desired, the behavior was rewarded, as described above.

Third step: Lifting the animal within the sling (Figure 1C). The pig was then left for 30 s and rewarded as mentioned above. Once rewarded, the animal was put back on the floor, rewarded again and the exercise was completed.

The frequency of these exercises was 4–5 times per week for 3 weeks and with no time limitation for each session.

In the case of the Almirall facility, the animals were used to the restraining sling from the very beginning since they arrived at the facility almost 5 months before this study. Briefly, 4 days after the arrival, the animals were approached by the caretakers, trying to establish contact and increase their confidence. Moreover, they were encouraged to stand on the weighing scale on their own. Treats (apple slices) and voices of encouragement were used as positive rewards. Sling training started 3–6 days later, when animals were confident with caretakers. Animals were lifted directly into the sling, and needed 5–8 training sessions to achieve what we considered complete habituation (the animal stayed on the sling in a relaxed manner). From that achievement on, training sessions were performed once per week, and the pigs always showed a complete habituated behavior.

### 2.3. Experimental Procedure and Saliva Collection

The assessment of stress levels in the sling was carried out by measuring cortisol levels in saliva samples. At CREBA facility, the procedure was performed one week after the end of the training described above. At Almirall facility, it was about five months after the initial training and one week after the last training session.

Five saliva samples were collected from each animal over 4 days. For this purpose, a swab (Salivabio Children Swaps, Salimetrics, Carlsbad, CA, USA) was introduced into the animal’s mouth, through a lateral corner, and the animal was allowed to chew for about 30 s. A kit of tubes, from the same manufacturer (Swab Storage Tube), were used, and a small tube was inserted into a larger outer tube and closed with a pressure cap. The wet swab was placed in the small tube for subsequent centrifugation and saliva collection.

**Figure 2:**
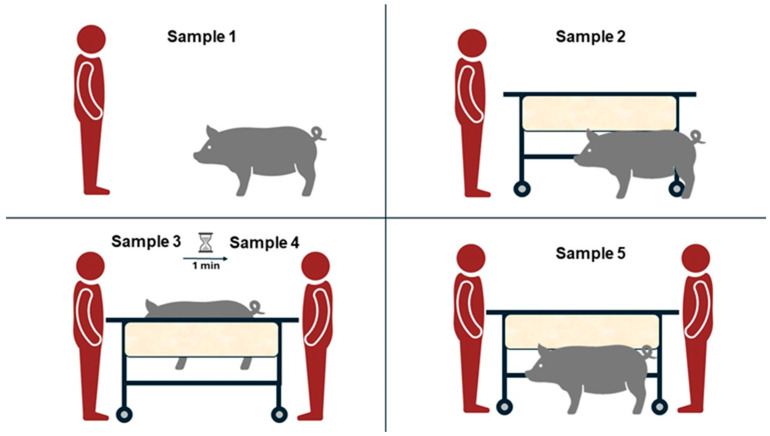
Schematic representation of the different sample moments. Sample 1: basal sample; Sample 2: sample obtained when the animal saw the sling; Sample 3: first sample obtained when the animal is put into the sling; Sample 4: second sample taken when animal is in the sling, one minute later; Sample 5: sample extracted after lower the animal from the restraint sling.

The sampling was always performed in the morning (between 08:00 and 12:00 a.m.), similar to the previous habituation protocol and according to the following sequence (Figure 2):

- Sample 1 (basal sample; S1): The technicians came into the pen and waited for 30 s for the animal to perceive their presence. Then, the technician introduced the swab between the lips of the animal, allowing mastication (Figure 1D).
- Sample 2 (S2): The technicians introduced the sling into the pen and waited for 30 s, checking that the pig had seen it. Afterward, the swab was introduced as explained above.
- Sample 3 (S3): The technicians put the animal in the sling, and thereafter the swab was inserted into its mouth (Figure 1C).
- Sample 4 (S4): Keeping the animal in the restraint sling, a second sample was taken 1 min after the previous one.
- Sample 5 (M5): After lowering the animal from the restraint sling, the pig was released on the floor, and the last sample was taken.

The tubes containing the wet swab were at room temperature until centrifugation (10 min at 1500× *g*) within 3 h after sampling. When this centrifugation was not enough to extract the saliva from the swab, an additional centrifugation was performed for 10 min at 1790× *g*.

Saliva samples were frozen at −20 °C (during a maximum of 90 days) until laboratory analyses.

Analyses for quantification of cortisol in saliva samples were performed at the “Laboratory for Hormone Analysis and Indicators of Stress, Animal Welfare and Reproduction” (LAIHA) of the Autonomous University of Barcelona (UAB) by using a commercial Enzyme-Linked ImmunoSorbent Assay (ELISA) kit (Neogen Corporation^©^, Ayr, UK). The intra-assay coefficient of variation (CV) was 3.24%, and the inter-assay CV was 21.60%. Concentrations were expressed as ng cortisol/mL saliva.

### 2.4. Statistical Analysis

The Friedman test was used to detect differences across multiple determinations.

The Pearson correlation coefficient was calculated to measure the linear correlation between the time the animals remained in the sling and salivary cortisol levels.

Comparisons of means of cortisol levels between groups were performed by the *t*-test, using the Levene’s test to compare variances. In addition, we used the two-tailed Fisher’s test to compare the qualitative variables referring to the difficulty of introducing the animal to the sling and to obtain the saliva sample.

Two-tailed *p*-values of less than 0.05 were considered evidence of statistical significance for all the analyses. Unless otherwise indicated, results are presented as mean ± SD.

All analyses were performed by using two statistical tools: PSPP 2.0 (https://www.gnu.org/software/pspp/ (accessed on 18 September 2024)) and OpenEpi 3.01 (https://www.openepi.com/Menu/OE_Menu.htm (accessed on 18 September 2024)).

## 3. Results

### 3.1. Body Weight of Animals Included in the Study

The mean body weight of animals in group HYB-F was 30 ± 9 kg; in group MIN-F, it was 33 ± 2 kg, and in group MIN-M, it was 46 ± 5 kg.

### 3.2. Training for Habituation to the Restraining Sling

At the time of salivary sample collection, only the animals in the group housed at Almirall (MIN-M) had achieved complete habituation to the restraint sling, having passed all habituation steps six months before. The groups of animals used in CREBA showed a different success rate during the short training period: HYB-F group: 2 out of 12 pigs completed habituation (that is, were able to remain in the sling during 30 s); MIN-F group: 1 out of 4 pigs. Saliva sampling was performed even when the animals failed to complete the previous habituation procedure.

### 3.3. Behavior of Animals to Restraining and Sampling

During the study and due to the very different behavior of the animals when handled for restraint in the sling, we decided to classify the animals into “strugglers” and “non-strugglers” based on the levels of resistance and/or struggle they showed when lifted into the restrainer. Animals that offered strong resistance were included in the struggler group, while those that showed only slight resistance or no resistance were considered non-strugglers. The results of that scoring are shown in Table 1 (A). The highest rate of animals with strong resistance was found in the HYB-F group, with no statistically significant differences between groups.

In addition, in group HYB-F, some animals could not be restrained in the sling on at least 1 day of the 4 study days (Table 1 (B)). Specifically, one animal (1/12) could never be restrained; another pig (1/12) could not be restrained on two different days (the 2nd and the 4th), and three pigs (3/12) could not be restrained on one day (the 2nd, 4th, and 4th). In these cases, when animals were not lifted into the sling, only S1 and S2 salivary samples were analyzed. All animals in the groups of miniature pigs could be restrained all the days.

With respect to saliva sampling, the number of samples successfully obtained in each group are shown in Table 1 (C). In hybrid pigs, only 80.3% of the samples were successfully obtained, while in miniature pigs, the percentage exceeded 96%.

In the HYB-F group, the sampling success rate in S3 + S4 was lower than in the basal sampling (S1), and this difference was statistically significant (*p* = 0.01). The comparison of failure rates between other pairs of samplings was not statistically significant. The overall percentage of sampling success in group HYB-F was similar between “struggler” and “non-struggler” pigs (81.8% and 78.9%, respectively). However, in the “struggler” animals, failures mainly occurred while the animals were restrained (S3 + S4). Specifically, 44.1% of such samples (15/34) were unsuccessful; meanwhile, the percentage rate for “non-struggler” pigs was 20.5% (9/44). That difference was statistically significant (*p* = 0.002).

### 3.4. Effects of the Restraint Sling on Salivary Cortisol Levels

In animals in the HYB-F group, the levels of salivary cortisol ranged from 1.34 to 8.79 ng/mL throughout the study. In group MIN-F, they ranged from 1.12 to 8.49 ng/mL and in group MIN-M, from 0.82 to 3.74 ng/mL (Table 2).

- Effect of the time of sampling (S1 to S5) on salivary cortisol levels.

Friedman’s test for the five repeated measures showed no statistical difference between the cortisol levels detected from S1 to S5 on any day and in any group. Furthermore, pooling all days, there was also no statistical correlation between cortisol levels in S1 to S5 in any group.

In the group of hybrid pigs, there were three animals with slightly higher basal cortisol levels than the others. Specifically, the mean of cortisol levels in S1 from those animals was 4.26 ± 1.38 ng/mL, whereas the mean for the whole group was 3.14 ± 1.52 ng/mL. To elucidate whether animals with such high basal levels might mask any change, we repeated the statistical analysis by deleting those three animals. Under these conditions, there was also no statistical difference in cortisol levels between sample times.

**Figure 3:**
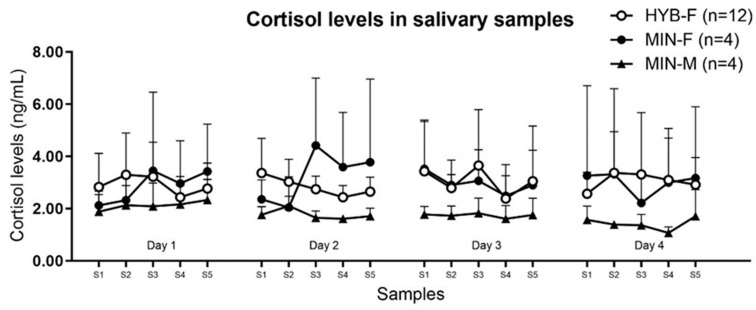
Graphical representation of the assessment of cortisol levels (ng/mL) in the five saliva samples collected during 4 consecutive days in the different groups. The meaning of the abbreviations S1 to S5 is described in the text (Section 2). HYB-F: hybrid female; MIN-F: miniature female; MIN-M: miniature male. Data are expressed as mean ± SD. Only above SD bars are depicted for clarity. Each point is the mean of n animals.

- Effect of the day of the study (days 1 to 4) on salivary cortisol levels (Figure 3).

When salivary cortisol levels were compared between consecutive days of the procedure within each group, there were no statistically significant differences. In other words, salivary cortisol levels did not significantly change across days in either group. However, in group MIN-M, cortisol levels in S5 tended to decrease over the days with a *p*-value close to statistical significance (*p* = 0.058).

- Influence of temperament on salivary cortisol levels.

**Figure 4:**
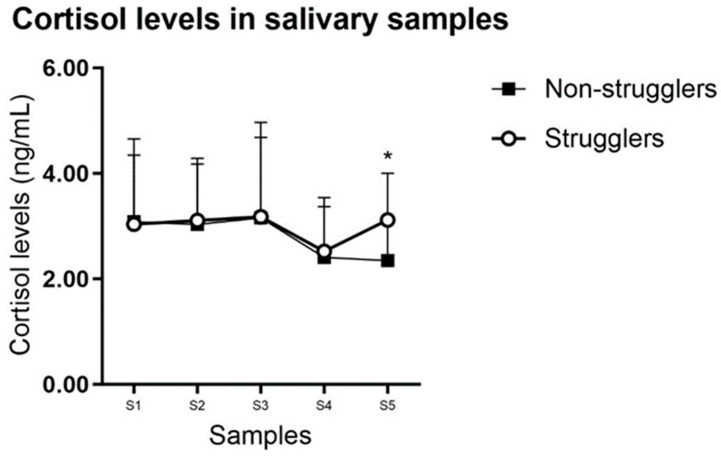
Graphical representation of the assessment of cortisol levels (ng/mL) in the five saliva samples collected during 4 consecutive days in the hybrid animal group (HYB-F) depending on the individual temperament (strugglers: animals which showed strong resistance; non-strugglers: animals showing only slight resistance or no resistance at all). The meaning of the abbreviations S1 to S5 is described in the text (Section 2). Data are expressed as mean ± SD. Only above SD bars are depicted for clarity. Each point is the mean of 13–26 samples. *: statistically significant difference (*p* < 0.05).

In hybrid pigs, salivary cortisol levels were similar between struggler and non-struggler pigs at the different times of manipulation, except for S5, in which cortisol levels were lower in non-struggler pigs (*p* = 0.002; Table 3 and Figure 4). In the struggler animals, a sampling of S1, S2, and S4 could not always be performed at the established time described in Section 2, but 1–2 min later. This did not statistically influence cortisol levels.

- Influence of restraint time on salivary cortisol levels.

The mean of restraint time in hybrid pigs was 3.30 ± 1.25 min, ranging from 2 to 7 min. Pearson’s test showed a statistical positive correlation between restraint time and cortisol levels in samples S1 (*p* = 0.001), S2 (*p* = 0.001), S3 (*p* = 0.003), and S4 (*p* = 0.003) but not in S5 (*p* = 0.7).

In group MIN-F, it was 3.53 ± 1.05 min, ranging from 2 to 5.20 min. There was no statistical correlation with cortisol levels in S4 nor in S5.

In group MIN-M, the restraint time was not recorded.

- Influence of pig strain on salivary cortisol levels.

A comparison of salivary cortisol levels between hybrid and miniature female pigs, manipulated in the same facility (HYB-F vs. MIN-F groups) did not give any statistically significant difference at any sampling time of the manipulation, irrespective of the day

## 4. Discussion

The use of cortisol as a biomarker of stress responses in pigs is a widely accepted procedure used in animal welfare studies and in studies directly related to stress pathophysiology [3,5,9]. Detection of this biomarker in saliva is particularly useful in situations where blood collection might interfere with cortisol values due to the potential stressfulness of the maneuver. Moreover, sampling can be carried out multiple times with fewer detrimental effects on the integrity of the pig [10]. The measurement of cortisol in pig saliva has been used in several procedures related to intensive swine farming: during transport [11] or housing (reviewed in [12]), in situations of aggression between males or other social challenges [13,14], in breeding-related processes [15,16], among others.

The goal of our study was to analyze the levels of salivary cortisol in pigs at different moments of a procedure for restraining into a sling, trying to detect any increase from the baseline levels which might indicate a stressful situation. In fact, although a hammock sling appears to be a refined way to restrain animals for brief manipulations (particularly if we compare it to others such as restraint loop), there are some factors that might be potentially stressful. For example, pigs do not like to have their limbs suspended in the air, probably because this is a very unnatural position for them, and their movements in the hammock are aimed at pulling their forelimbs out of the openings and tucking them under the body, in a way in which they seem to be more comfortable. In addition, there were high increases in heart rate and blood pressure levels when the pigs are suspended in a harness with their feet lifted off the ground [17].

We used two different pig strains (both hybrid and miniature pigs) housed in two different facilities, in which the training and habituation of the pigs to different husbandry procedures had different approaches. In one of the facilities (Almirall), only miniature pigs were utilized (group MIN-M), which were frequently handled to be restrained, so that at the beginning of this study, the animals were completely used to the sling. In fact, they did not struggle and stayed completely calm, chewing the swabs used for collecting saliva samples.

On the other hand, the pigs in the CREBA facility were hybrid or miniature (groups HYB-F and MIN-F, respectively) and were just trained several weeks before the study. Despite the fact that the saliva sampling was not an invasive or painful maneuver and most of the hybrid animals were reluctant about the sling and even struggled, it was not possible to obtain saliva samples in some cases. In fact, sampling failure rates were significantly higher in hybrid pigs than in miniature ones. In addition, the rates of unsuccessful saliva collection in hybrid pigs were higher when the animals were restrained in the sling, which was not seen in the miniature pigs.

In general, individual factors must always be considered when working with pigs, for example, temperament, motivation, and what researchers know as “coping style”, which is an individual adaptive strategy that describes the animal’s response to its environment in terms of reducing the effect of aversive stimuli [18,19]. All these factors, among others, influence the personality of the individual animals and might mask the cause of differences among the experimental groups [20]. According to the “coping style” hypothesis, animals can be classified as reactive or proactive by a behavioral test called “backtest” [21]. Proactive animals tend to be more aggressive, dominant, and less flexible to a changing environment, whereas reactive animals are just the opposite [21,22]. However, results of a later study did not support the theory that backtest performance is indicative of behavior in a test of aggression or a test of fear/curiosity in response to novelty [23].

In the present study, we did not perform any behavioral test; however, we observed two patterns of behavior of animals when they were handled for restraining, which led us to classify them into “strugglers” or “non-strugglers”. The animals classified as “strugglers” offered high physical resistance to being lifted into the sling and to saliva sampling. The “non-strugglers” were handled without strong resistance, and the cotton swab was easily introduced into their mouth. This difference in temperament was particularly evident in hybrid animals, since half of them (6/12) were considered as strugglers. Moreover, five of them were impossible to restrain on one or more days due to strong resistance and struggling behavior. Interestingly, all the miniature pigs showed no or slight resistance, independently of the facility, so they were all considered as “non-strugglers”. The weight of the animals was excluded as a possible factor influencing the discomfort, because the mean body weight of the hybrid group was significantly lower than that of the male miniature group (30 ± 9 vs. 46 ± 5 kg; *p* = 0.005). However, it was not possible for us to ascertain if differences of behavior were related to individual temperaments, to the way of breeding and management on the farms of origin, or even to genetic differences.

A problem derived mainly from struggling behavior was the high rate of sampling failure when animals were restrained. Specifically, 44.1% of the saliva collection attempts at those moments failed to achieve the minimum volume needed for quantitative cortisol analysis (200 μL), even if the animal accepted the swab. As far as we know, there are no studies evaluating the amount of saliva secreted by pigs when confronted with a potential stressor like the sling restrainer, though the dry mouth or xerostomia could be related to acute stress in some species, including humans [24,25]. It is relevant to note that cortisol concentrations in saliva are independent of flow rate (reviewed in [1,6].

All the study samples were always collected in the morning (between 08:00 and 12:00) at the same time as the previous habituation protocol to reduce any circadian rhythm effect. Cortisol and other metabolites fluctuate according to a circadian pattern, and their levels are higher in the morning and lower in the afternoon and evening [26]. Some authors argue that the optimal time for sampling is around 6 p.m., because the cortisol baseline concentrations are at their lowest [5]. However, we considered it more appropriate to perform this study during the hours when animals are usually handled in an animal facility.

Apparent signs of stress and discomfort did not correlate with changes in salivary cortisol levels in our study. Some of the hybrid pigs showed very apparent signs of stress, discomfort, and even fear such as defecation, vocalization, skin redness, and attempts to jump out of the sling. However, cortisol levels in such hybrid animals classified as “strugglers” did not differ from those obtained in the “non-struggler” animals (Figure 4). This lack of correlation between such apparent signs of stress and salivary cortisol levels was an unexpected result of this study.

While this study was being performed, a pig at the CREBA facility (not enrolled in the present study) suffered an articular injury which was unsuccessfully treated for a couple of days. Salivary cortisol levels were measured; specifically, we took two samples separated by a few minutes and the levels obtained were 81.22 ng/mL and 109.12 ng/mL. Although coincidental, this case served as a positive control for both chronic and acute stresses, evidenced by both clinical symptomatology and laboratorial analysis procedure used in the study.

It could be argued that there was not enough time from the stressor stimulus (sling restraint) to elicit the presence of enough amount of cortisol to be detected in saliva. However, the literature on this field suggests that the cortisol reaches saliva in a few minutes, though depending on the intensity and duration of the stressor. Using a snare restraint, some authors reported an immediate or very fast increase (5 min) in the salivary cortisol levels of pigs restrained briefly with a snare [5,27]. Stimulation with ACTH followed by matched blood and saliva sampling determined that the lag time between the increases in total plasma cortisol and salivary cortisol was less than 2 min (reviewed in [1]). In the present study, the animals were always more than 2 min in the sling (specifically, between 2 and 7).

Interestingly, in the hybrid animals, the time spent in the sling was statistically correlated with cortisol levels, but this positive correlation was already observed before the animals were placed in the sling (from S1 to S4). In our opinion, this result suggests that animals with a higher basal cortisol level spent more time in the sling due to the difficulty of handling and obtaining salivary samples. By contrast, a longer restraint time did not imply higher cortisol levels, as levels at S5 were not correlated with restraint time.

One hypothesis to explain the stability of salivary cortisol levels, despite the apparent signs of stress, could be a misinterpretation of such signs, as it might not mean a stressful situation, but rather the animals’ knowledge that this was the way to end the discomfort, because they were highly socialized with the operators. In fact, when the animals were released, they remained calm and stayed close to them and to the sling structure, instead of running away.

## 5. Conclusions

In conclusion, in female hybrid farm pigs, female miniature pigs, and castrated male miniature pigs, salivary cortisol levels did not increase when the pigs were lifted and briefly restrained in a sling, although some of them showed apparent signs of stress and fear. The lack of correlation between such apparent stress and cortisol levels might be because there was no real stress and, rather, the animals were in a familiar environment looking for a way to release themselves from a known discomfort. In light of this unexpected conclusion, further studies are needed to collect other physiological and behavioral data to clarify what actually happens when pigs are restrained in a sling.

## Figures and Tables

**Table 1:** (**A**) Results of the evaluation of the struggling behavior of the animals when restrained in the sling. (**B**) Summary of the animals who were able to be lifted into the sling. (**C**) Summary of samples obtained and sampling success rates.

**A**	**Number of Animals**						
**Group**	**Non-Strugglers**			**Strugglers**			
HYB-F (n = 12)	6			6			
MIN-F (n = 4)	4			0			
MIN-M (n = 4)	4			0			
**B**	**Number of Animals**						
**Group**	**Never Lifted**	**Not Lifted on 2 days**		**Not Lifted on 1 day**		**Always Lifted**	
HYB-F (n = 12)	1	1		3		7	
MIN-F (n = 4)	0	0		0		4	
MIN-M (n = 4)	0	0		0		4	
**C**		**Salivary Samples**					
**Group**		**S1**	**S2**	**S3 + S4**	**S5**	**Total**	**% of Success**
HYB-F	Total sampling attempts	48	48	78	39	213	80.3%
	Samples obtained	43	44	54	30	171	
MIN-F	Total sampling attempts	20	20	20	20	80	97.5%
	Samples obtained	20	19	20	19	78	
MIN-M	Total sampling attempts	20	20	20	20	80	96.2%
	Samples obtained	19	20	18	20	77	

**Table 2:** Mean values and ranges of cortisol levels detected in saliva samples, grouped by the time of sample collection in relation to the restraining of the animals in the sling. The meaning of the abbreviations S1 to S5 is described in the text (Section 2).

	Cortisol Levels (ng/mL)									
	S1		S2		S3		S4		S5	
Groups	Mean	s.d.	Mean	s.d.	Mean	s.d.	Mean	s.d.	Mean	s.d.
	Range		Range		Range		Range		Range	
HYB-F	3.14	1.52	3.12	1.20	3.29	1.72	2.56	1.12	2.87	0.95
	1.53–7.87		1.64–6.09		1.68–8.79		1.34–5.91		1.51–5.51	
MIN-F	2.82	1.89	2.69	1.71	3.29	2.07	3.01	1.56	3.31	2.33
	1.12–8.39		1.33–8.21		1.29–7.96		1.38–6.47		1.43–8.49	
MIN-M	1.75	0.45	1.84	0.70	1.71	0.55	1.65	0.57	1.88	0.71
	1.19–2.80		1.18–3.74		0.84–3.12		0.82–3.08		1.07–3.48	

**Table 3:** Mean values and ranges of cortisol levels detected in saliva samples from hybrid animals grouped by behavior during the procedure (strugglers and non-strugglers). The meaning of the abbreviations S1 to S5 is described in the text (Section 2).

	Cortisol Levels (ng/mL)									
	S1		S2		S3		S4		S5	
Animals	Mean	s.d.	Mean	s.d.	Mean	s.d.	Mean	s.d.	Mean	s.d.
	Range		Range		Range		Range		Range	
Strugglers	3.04	1.31	3.11	1.18	3.18	1.79	2.52	0.85	3.12	0.88
	1.56–6.57		1.64–5.7		1.83–8.79		1.63–5.15		2.01–5.51	
Non-Strugglers	3.08	1.57	3.03	1,15	3.16	1.52	2.41	1.14	2.35	0.75
	1.53–7.87		1.82–6.09		1.68–7.44		1.34–5.91		1.51–3.96	

## Data Availability

The original contributions presented in the study are included in the article, and further inquiries can be directed to the corresponding author.
